# Effects of Dietary Eicosapentaenoic Acid (EPA) Supplementation in High-Fat Fed Mice on Lipid Metabolism and Apelin/APJ System in Skeletal Muscle

**DOI:** 10.1371/journal.pone.0078874

**Published:** 2013-11-07

**Authors:** Chantal Bertrand, Angelica Pignalosa, Estelle Wanecq, Chloé Rancoule, Aurélie Batut, Simon Deleruyelle, Lillà Lionetti, Philippe Valet, Isabelle Castan-Laurell

**Affiliations:** 1 Institut National de la Santé et de la Recherche Médicale (INSERM), U1048, Toulouse, Cedex 4, France; 2 Université de Toulouse, UPS, Institut des Maladies Métaboliques et Cardiovasculaires, Toulouse, France; 3 Universitat degli Studi “Federico II”, Napoli, Italy; State University of Rio de Janeiro, Biomedical Center, Institute of Biology, Brazil

## Abstract

Various studies have shown that eicosapentaenoic acid (EPA) has beneficial effects on obesity and associated disorders. Apelin, the ligand of APJ receptor also exerts insulin-sensitizing effects especially by improving muscle metabolism. EPA has been shown to increase apelin production in adipose tissue but its effects in muscle have not been addressed. Thus, the effects of EPA supplementation (36 g/kg EPA) in high-fat diet (HFD) (45% fat, 20% protein, 35% carbohydrate) were studied in mice with focus on muscle lipid metabolism and apelin/APJ expression. Compared with HFD mice, HFD+EPA mice had significantly less weight gain, fat mass, lower blood glucose, insulinemia and hepatic steatosis after 10 weeks of diet. In addition, EPA prevented muscle metabolism alterations since intramuscular triglycerides were decreased and β-oxidation increased. In soleus muscles of HFD+EPA mice, apelin and APJ expression were significantly increased compared to HFD mice. However, plasma apelin concentrations in HFD and HFD+EPA mice were similar. EPA-induced apelin expression was confirmed in differentiated C2C12 myocytes but in this model, apelin secretion was also increased in response to EPA treatment. In conclusion, EPA supplementation in HFD prevents obesity and metabolic alterations in mice, especially in skeletal muscle. Since EPA increases apelin/APJ expression in muscle, apelin may act in a paracrine/autocrine manner to contribute to these benefical effects.

## Introduction

Obesity and associated diseases such as type 2 diabetes or hypertension are a major public health problem. Different strategies, from lifestyle changes to pharmacological interventions, have been shown to be successful in improving metabolism and reducing weight gain. The role of the long-chain omega-3 polyinsaturated fatty acids (n-3 PUFAs) has been largely documented. In clinical trials, the delay of the onset of cardiovascular events and a decrease in obesity and in the incidence of type 2 diabetes has been described in response to dietary intake of PUFAs [1], [2], [3]. However, change in insulin sensitivity or body weight after n-3 PUFAs supplementation has not always been demonstrated [4]. Eicosapentaenoic acid (EPA), one of the major PUFAs contained in fish oil has been shown to exert anti-atherogenic, anti-inflammatory and lipid-lowering effects [4], [5]. In numerous animal models, EPA supplementation into high-fat diets (HFD) has also been shown to prevent [6]–[9] but also to reverse obesity [6]. Since adipose tissue is now considered as an endocrine organ, different studies have also demonstrated the effect of EPA (alone or in combination with other n-3 PUFAs) on different adipokines expression and/or secretion. EPA was shown to increase leptin expression and secretion in cultured adipocytes [10], [11] but not in vivo [12]. Increased adiponectin has been observed in adipose tissue of ob/ob mice receiving n-3 PUFA-enriched diet [9] or in adipose tissue of HFD fed mice [13] and in cultured human adipocytes in response to EPA [14]. Due to the more recent identification of apelin as an adipokine involved in glucose metabolism [15], EPA alone has also been shown to increase apelin expression and secretion in 3T3-L1 adipocytes [16] and in adipose tissue of cafeteria diet-fed rats [17].

In addition, apelin is also a peptide expressed in different tissues such as skeletal muscles [18], [19]. The apelin receptor, APJ, is also often expressed in the same tissues than apelin. Insulin is able to regulate both apelin and APJ expression in skeletal muscles [19]. In obese and insulin-resistant mice, apelin and APJ expression are increased in adipose tissue but not in skeletal muscles [19]. Thus, there is a tissue-dependent regulation of the apelin/APJ system that could also be modified with the severity of insulin resistance (for example in diabetic db/db mice) [19]. More recently, apelin treatment in obese and insulin resistant mice has been shown to reduce fat mass and to improve muscle metabolism by increasing fatty acid oxidation and mitochondrial biogenesis [20]. Prevention of diet-induced obesity and increased oxidative capacities in muscles has also been found in transgenic mice overexpressing apelin [21]. Thus, apelin is considered as an insulin-sensitizing factor.

Since EPA and apelin share common metabolic features, the aim of this work was to test the hypothesis that the beneficial effects of EPA could be in part due to an up-regulation of the apelin/APJ system. Since apelin regulation has been studied in adipose tissue, we focused on muscle metabolism and on expression of both apelin and APJ in skeletal muscle of mice fed with a HFD supplemented with EPA. Moreover, only the effect of EPA was studied in order to compare with previous studies on apelin expression and on metabolic effects in mice. Dietary EPA supplementation to HFD prevented alterations in muscle lipid metabolism induced by HFD. Moreover, it increased significantly apelin/APJ expression in soleus compared to HFD fed mice. In vitro, in differentiated C2C12 myocytes, EPA also up-regulated apelin expression and its secretion suggesting that EPA has a direct effect on muscle apelin expression and that apelin could act as a paracrine/autocrine factor.

## Materials and Methods

### Animals and diets

Mice were handled in accordance with the principles and guidelines established by the Institut National de la Santé et de la Recherche Médicale (INSERM), permission number C31 55507. The animal protocol was approved by the animal ethics committee of the unit US006 CREFRE (Centre Régional d'Exploration Fonctionnelle et Ressources Expérimentales).

Nine-week-old male C57Bl6/J mice were purchased from Charles River Laboratories (l'Arbresle, France). Mice were housed conventionally in a constant temperature (20–22°C) and humidity (50–60%) animal room, with a 12/12 h light/dark cycle (lights on at 7:00 A.M.) and free access to food and water. Mice were fed a normal diet (ND), (10, 20 and 70% of energy from fat, protein and carbohydrate respectively) or a HFD containing 45% fat, 20% protein, 35% carbohydrate, or HFD containing EPA (HFD+EPA) 45% fat including 3.6% EPA Ethyl Ester (36 g/kg wt/wt), 20% protein, 35% carbohydrate. Both HFD were containing vitamin E (0.13%) to prevent peroxidation of EPA. EPA (99.3% purity) was kindly provided by KD Pharma, Bexbach Germany. All diets were custom diets made by Research Diets (Research Diets Inc, New Brunswick, NJ, USA). After 10 weeks of diet, all mice were sacrificed after a 6-hour fast.

### Plasma parameters

Plasma insulin and apelin concentrations were determined with an ultrasensitive mouse insulin ELISA kit (Mercodia, Uppsala, Sweden) and a nonselective apelin-12 EIA kit (Phoenix Pharmaceuticals, Belmont, CA), respectively. Glycemia was measured with a glucometer (Accu-check, Roche Diagnostics Grenoble, France) on blood from the tail vein in fasted mice. EPA and long-chain fatty acids levels especially, were measured by HPLC after methylation. Briefly, plasma (10 µl) was hydrolysed in 1 ml KOH (0.5 M in methanol) at 50°C for 30 minutes, in the presence of the internal standards glyceryl triheptadecanoate (2 mg), and transmethylated in boron trifluoride methanol solution 14% (1 ml) and hexane (1 ml) at 80°C for 1 h. After addition of water to the crude, fatty acid methyl esters (FAMEs) were extracted with hexane, brought to dryness and dissolved in ethyl acetate (20 ml). FAMEs were analyzed by gas-liquid chromatography [22] on a Clarus 600 Perkin Elmer system using a Famewax RESTEK fused silica capillary columns (30 m×0.32 mm i.d, 0.25 mm film thickness). Oven temperature was programmed from 110°C to 220°C at a rate of 2°C per min and the carrier gas was hydrogen (0.5 bar). The injector and the detector were at 225°C and 245°C respectively.

### Food intake measurement

Food intake was measured 2 days and 6 weeks after the beginning of the different diets. Mice were housed individually on a 12 cm-diameter grid of metabolic cages (BioSeb, Spain). They were given free access to food and water, and the food was weighed every 12 hours during 48 hours, after 24 h of acclimatization.

### Body fat mass composition

To determine fat and lean mass, mice were placed in a clear plastic holder, without anesthesia or sedation, and inserted into the EchoMRI-3-in-1 system (Echo Medical Systems, Houston, TX). Total body fat and lean mass were measured one day before sacrifice.

### Oral glucose tolerance test (OGTT)

OGTT was performed one week before the end of the protocol. After 6 h fast during the light period, mice received glucose (3 g/kg body weight) by gavage through a gastric tube (outer diameter 1.2 mm). Glycemia was monitored from the tail vein 0, 15, 30, 45, 60, 90, and 120 min after glucose administration, using a glucometer (Accu-check, Roche Diagnostic, Grenoble, France). The area under the curve (AUC) was calculated.

### Palmitate oxidation

Palmitate oxidation was determined ex vivo in whole soleus muscle as previously described [20]. Briefly, muscles were incubated in modified Krebs-Henseleit buffer containing 1.5% FA-free BSA, 5 mmol/L glucose, 1 mmol/L palmitate, and 0.5 µCi/mL ^14^C-palmitate (PerkinElmer) at 37°C for 60 min in a sealed glass vial. At the end of the incubation, tissues were removed and homogenized in 800 µL lysis buffer for protein quantification by the method of Bradford (BioRad, France). Complete oxidation was determined by acidifying the incubation buffer with 1 mL of 1 mol/L H2SO4, and the ^14^CO_2_ was trapped by benzethonium hydroxide (Sigma-Aldrich, St. Louis, MO, USA) placed in a 0.5 mL microtube in the vial. After 120 min, the microtube was removed and placed in a scintillation vial, and the radioactivity was counted (Cytoscint, MP Biomedicals).

### Measurement of liver and skeletal muscle lipid content and liver histological analysis

Gastrocnemius muscle (red part) and a sample of liver were used to extract total lipids by the Folch method [23], with minor modifications. Briefly, the frozen tissues were homogenized and the lipids were extracted using 2.5 ml chloroform/methanol (2/1: v/v) and 1.2 ml KCl/HCl 2N. After centrifugation, 100 µL of the organic phase, containing neutral lipids, was brought to dryness under nitrogen. For determination of triglycerides (TG) content, the lipid pellet was solubilized in isopropanol and TG concentrations were determined using the enzymatic TG PAP kit (Biomérieux, Marcy l'Etoile, France) following the manufacturer's instructions. Muscle TG content was normalized to protein content (Bradford method) and liver TG content was normalized to tissue weight. For histological analysis, liver samples were fixed in 4% buffered formalin for 24 hours, and maintained at 4°C in 70% ethanol. Fixed tissues were processed routinely for paraffin embedding, and 4-µm sections were prepared and stained with hematoxylin-eosin (H&E) and viewed using an optical microscope (Nikon Eclipse TE 2000-U).

### Gene expression study

Immediately after euthanasia, soleus muscle, adipose tissue (perigonadal and subcutaneous depots) and liver were taken and frozen in liquid nitrogen, and total RNAs were isolated using the GeneJET RNA Purification kit (Fermentas). Total RNAs (500 ng) were reverse transcribed using Superscript II reverse transcriptase (Invitrogen) in the presence of a random hexamer. The same reaction was performed without Superscript II to estimate DNA contamination. Real time PCR was performed as previously described [20]. Analysis of HPRT expression was performed to normalize gene expression. The sequences of the primers for apelin are: GTTTGTGGAGTGCCACTG (forward) and CGAAGTTCTGGGCTTCAC (reverse), for APJ: GCTGTGCCTGTCATGGTGTT (forward) and CACTGGATCTTGGTGCCATTT (reverse), for leptin: GGGCTTCACCCCATTCTGA (forward) and TGGCTATCTGCAGCACATTTTG (reverse), for adiponectin: TGGAATGACAGGAGCTGAAGG (forward) and TATAAGCGGCTTCTCCAGGCT (reverse), for SREBP-1c: CTGGCTTGGTGATGCTATGTTG (forward) and GACCATCAAGGCCCCTCAA (reverse), for UCP3: GCTGGAGTCTCACCTGTTTACTG (forward) and ACAGAAGCCAGCTCCAAAGG (reverse); for CTP1b: GTGCAAGCAGCCCGTCTAG (forward) and TTGCGGCGATACATGATCA (reverse), and for the housekeeping gene HPRT: TGGCCATCTGCCTAGTAAAGC (forward) and GGACGCAGCAACTGACATTTC (reverse).

### Cell culture experiments

C2C12 mouse myoblasts (ATCC number CRL-1772™) were cultured at 37°C in DMEM 1 g/L glucose (Sigma-Aldrich), supplemented with 20% FBS, 292 mg/ml glutamine and antibiotics (2.5 µg/ml amphotericin and 50 µg/ml gentamicin) in 12-well culture plates. To induce differentiation after confluence, medium was changed to DMEM 4.5 g/L glucose, supplemented with 5% horse serum. Cells were maintained for 14 days in order to obtain differentiated polynucleated myotubes. Prior to treatment with EPA (Sigma-Aldrich), cells were serum-deprived for 12 hours. BSA and 200 µM EPA (1∶5) were mixed thoroughly with serum-free medium the day before the stimulation, from a 10 mM EPA stock solution in ethanol, and kept at 4°C overnight. Equivalent volume of ethanol was mixed with BSA for the control condition. Different EPA concentrations (10, 50, 100 and 200 µM) were prepared from the 200 µM solution, and 1 ml was added to the cells. After 24 h, the medium was collected and kept at −80°C and the cells were washed with ice-cold PBS, and harvested in lysis buffer (Fermentas) containing β-mercapto-ethanol for RNA extraction. Apelin concentration in the medium was measured as described above after addition of 0.2 TIU/ml Aprotinin (Sigma-Aldrich) in the medium and concentration of the medium by evaporation (Concentrator 5301, Eppendorf). Differentiated C2C12 cells were also treated after 12-hour serum deprivation with the PI3K inhibitor LY 294002 (20 µM) (Sigma-Aldrich) or the ERK 1/2 inhibitor U0126 (20 µM) (Cell Signaling) alone or in the presence of 100 µM EPA for 24 h. The inhibitors were added 30 min prior to the addition of EPA.

### Statistical analysis

Data were expressed as means ±SEM. Statistical analyses were performed with GraphPad Prism 5.0 software (GraphPad Software, San Diego, CA). Analysis of differences between groups was performed with one-way ANOVA followed by Tukey test *post hoc*. Non parametric Student t test was also used when appropriate. Differences were considered significant at P<0.05.

## Results

### Bioavailability of EPA and fatty acid composition in plasma of ND, HFD and HFD+EPA mice

**Figure 1:**
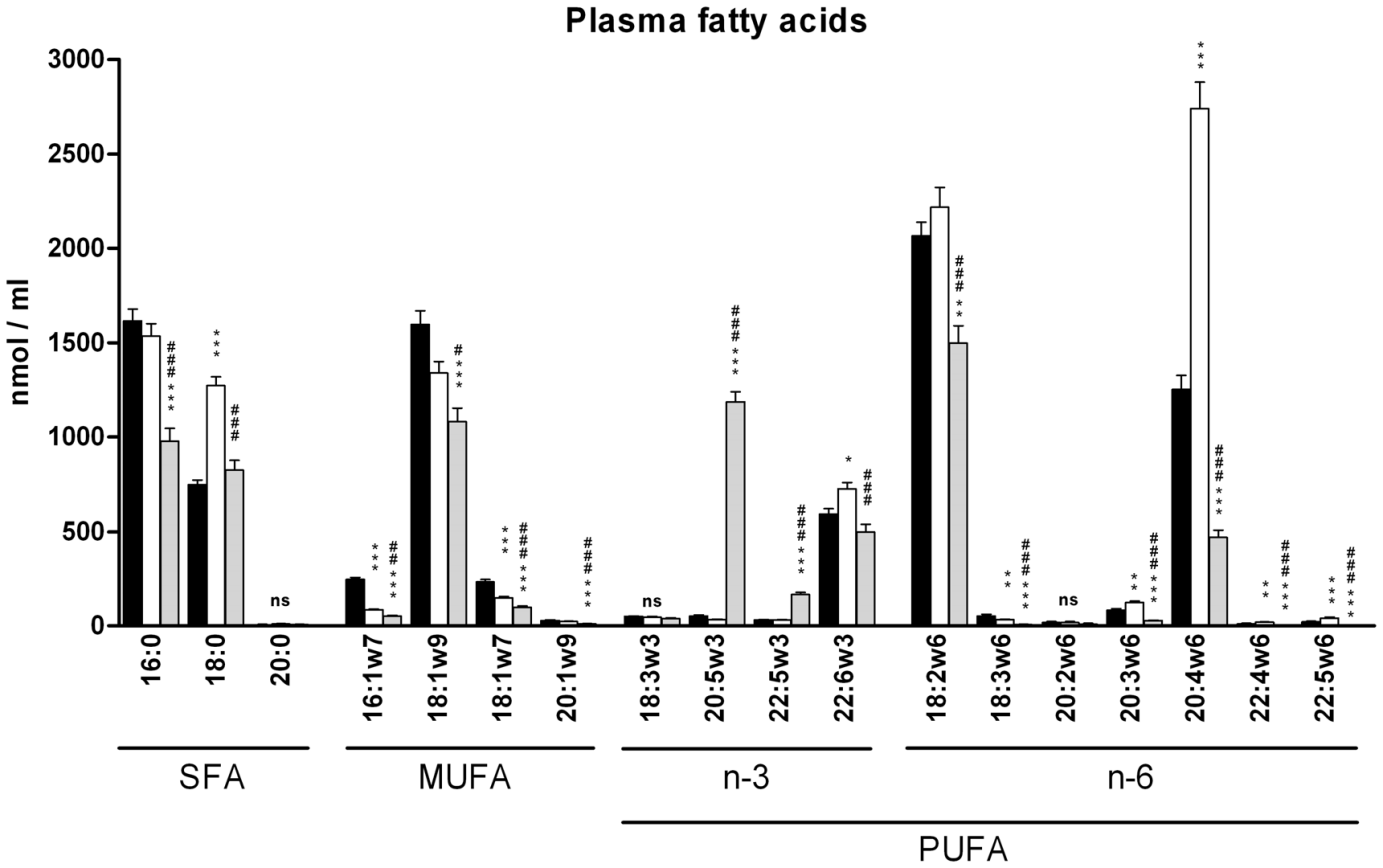
Plasma lipid composition. SFA (Saturated Fatty Acids), MUFAs (Mono-Unsaturated Fatty Acids) and PUFAs (Poly-Unsaturated Fatty Acids) were measured by gas-liquid chromatography in plasma of fasted mice after 10 weeks feeding with ND (black column, n = 6), HFD (white column, n = 12) or HFD+EPA (grey column, n = 9). Data are expressed as mean ±SEM. *p<0.05, **p<0.01, ***p<0.001 vs ND; # p<0.05, ## p<0.01, ### p<0.001 vs HFD. ns: not significant

First, detectable plasma lipids were analyzed in the three groups of mice after 10-week feeding, in order to quantify the increment in EPA concentration especially in the HFD+EPA group. As shown in Fig. 1, EPA (C20:5 n-3) was significantly increased in plasma of HFD+EPA compared to ND and HFD mice: 53.5±4.3 µM in ND vs 34.1±1.5 µM in HFD and 1225.5±50.1 µM in HFD+EPA. Interestingly, PUFAs from the omega-6 family, saturated and mono unsaturated fatty acids were globally significantly decreased in plasma of HFD+EPA mice compared to HFD mice. Thus, arachidonic acid-derived lipids, known to exert pro-inflammatory effects [6], [24], were decreased in plasma of HFD+EPA mice.

### Effect of EPA on HFD-induced obesity

**Figure 2:**
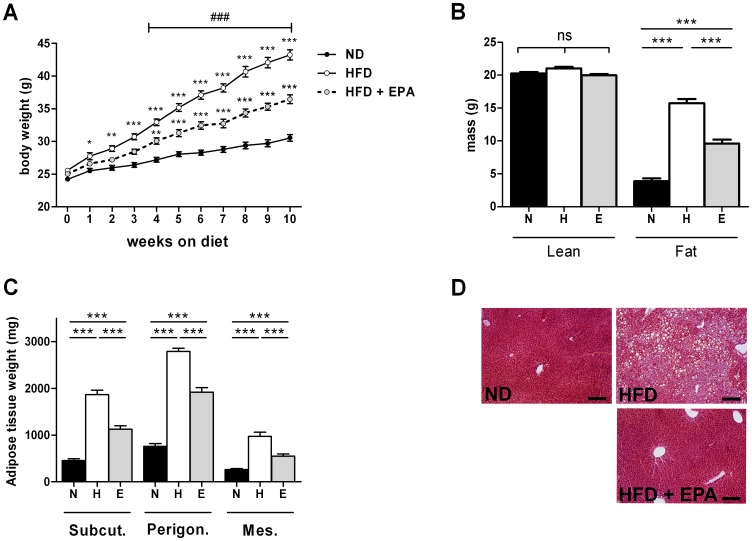
Protective effect of EPA supplementation on the HFD-induced obesity. (A) Body weight in mice after 10-week feeding with a ND (n = 12), HFD (n = 14) or HFD+EPA(n = 14). Results represent mean ±SEM. *p<0.05, **p<0.01, ***p<0.001 vs ND; and ### p<0.001 vs HFD, ns: not significant. (B) fat and lean mass and (C) fat pads weights of subcutaneous (Subcut), perigonadal (Perigon) and mesenteric (Mes) adipose tissue in mice fed for 10 weeks with a ND or N (n = 12), HFD or H (n = 14) or HFD+EPA or E (n = 14) Results represent mean ±SEM. ***p<0.001, (D) Representative photographs of H&E staining of liver section of mice fed the different diets for 10 weeks (bar = 200 µm).

**Figure 3:**
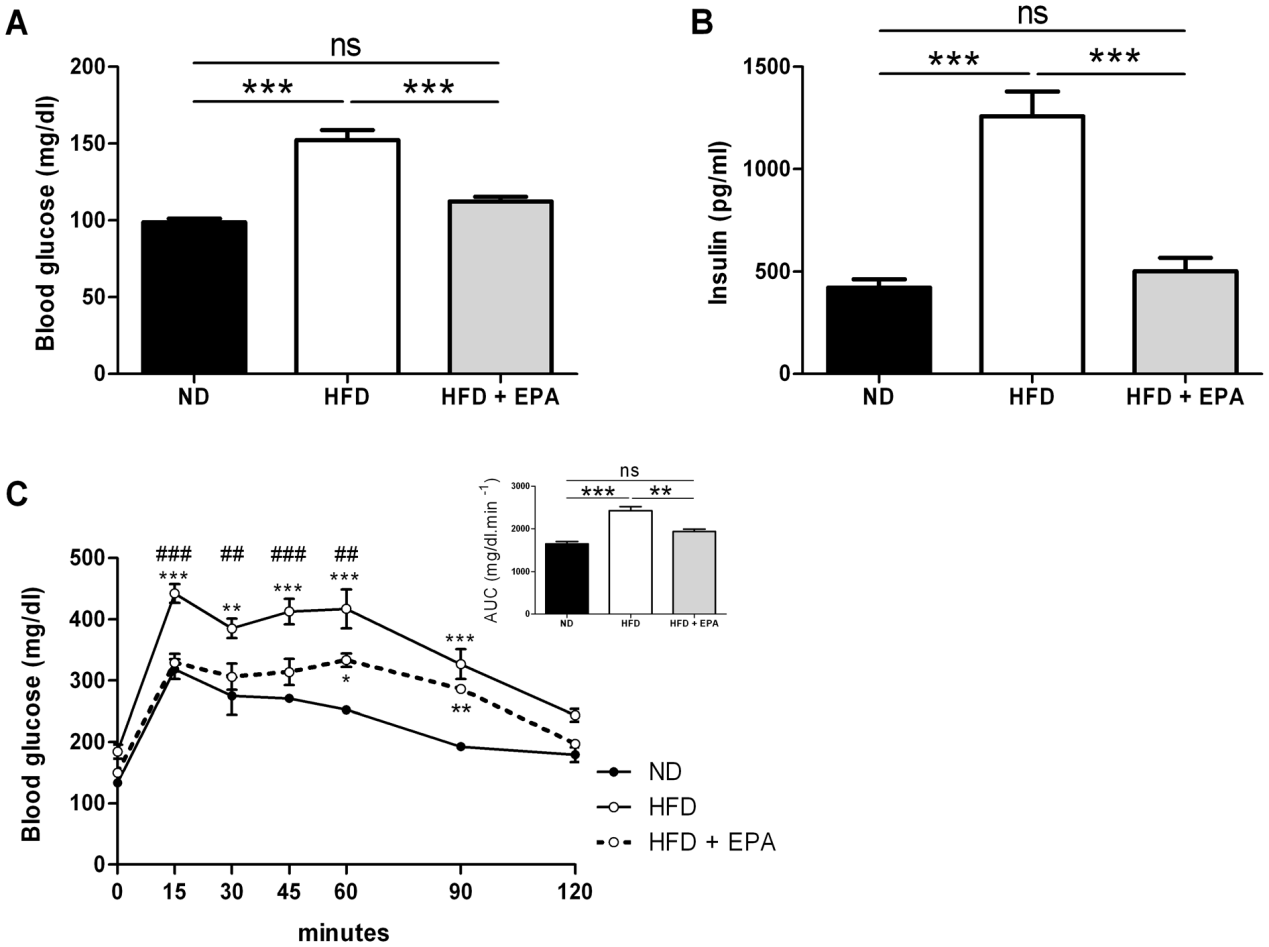
Protective effect of EPA supplementation on the HFD-induced impaired glucose metabolism. (A) Glycemia and (B) insulinemia in 6 h-fasted mice fed a ND (n = 12), a HFD (n = 14) or a HFD+EPA (n = 14) after 10 weeks. Results represent mean ±SEM. ***p<0.001, (C) OGTT curves and area under the curve (AUC) of glycemia monitored during OGTT performed on 6 h fasted (during light period) mice after 9 weeks of diet in ND (n = 4), HFD (n = 6) and HFD+EPA (n = 6) mice. Results represent mean ±SEM. *p<0.05, **p<0.01, ***p<0.001 vs ND; and ## p<0.01, ### p<0.001 vs HFD.

Both HFD and HFD+EPA groups gained weight compared to control ND mice (Fig. 2A) but to a significant lesser extent for HFD+EPA mice. After 10-week feeding, HFD+EPA mice had higher fat mass (Fig. 2B) and fat pads weights (Fig. 2C) than control ND mice but significantly less than HFD fed mice. However, food intake was not different between HFD and HFD+EPA mice 2 days after the beginning of the protocol (HFD: 15.5±1.0 kcal/24 h and HFD+EPA: 19.0±2.0 kcal/24 h) and after 6 weeks (HFD: 6.8±2.6 kcal/24 h and HFD+EPA: 7.7±2.1 kcal/24 h). Moreover, there was less lipid accumulation in the liver of HFD+EPA mice than in the liver of HFD mice (Fig. 2D). This was in agreement with i) the measurement of triglycerides content: 0.24±0.02 for ND (n = 10) vs 0.38±0.04 for HFD (n = 14) and 0.17***±0.02 g TG/g liver for HFD+EPA (n = 14) ***P<0.001 vs HFD and ii) the expression of hepatic sterol regulatory element-binding protein-1c (SREBP-1c), a transcription factor involved in lipid synthesis: ND (n = 10): 100±10.94 AU, HFD (n = 14) 246.2±38.7 AU vs 143.6*±19.8 AU in HFD+EPA (n = 14) *P<0.05 vs HFD. HFD+EPA mice also had a lower glycemia (Fig.3A) and insulinemia (Fig.3B) and a better glucose tolerance as shown in figure 3C compared to HFD fed mice. Thus, dietary EPA supplementation protected against weight gain, hepatic steatosis and insulin resistance.

### Effect of EPA on adipokines gene expression

**Figure 4:**
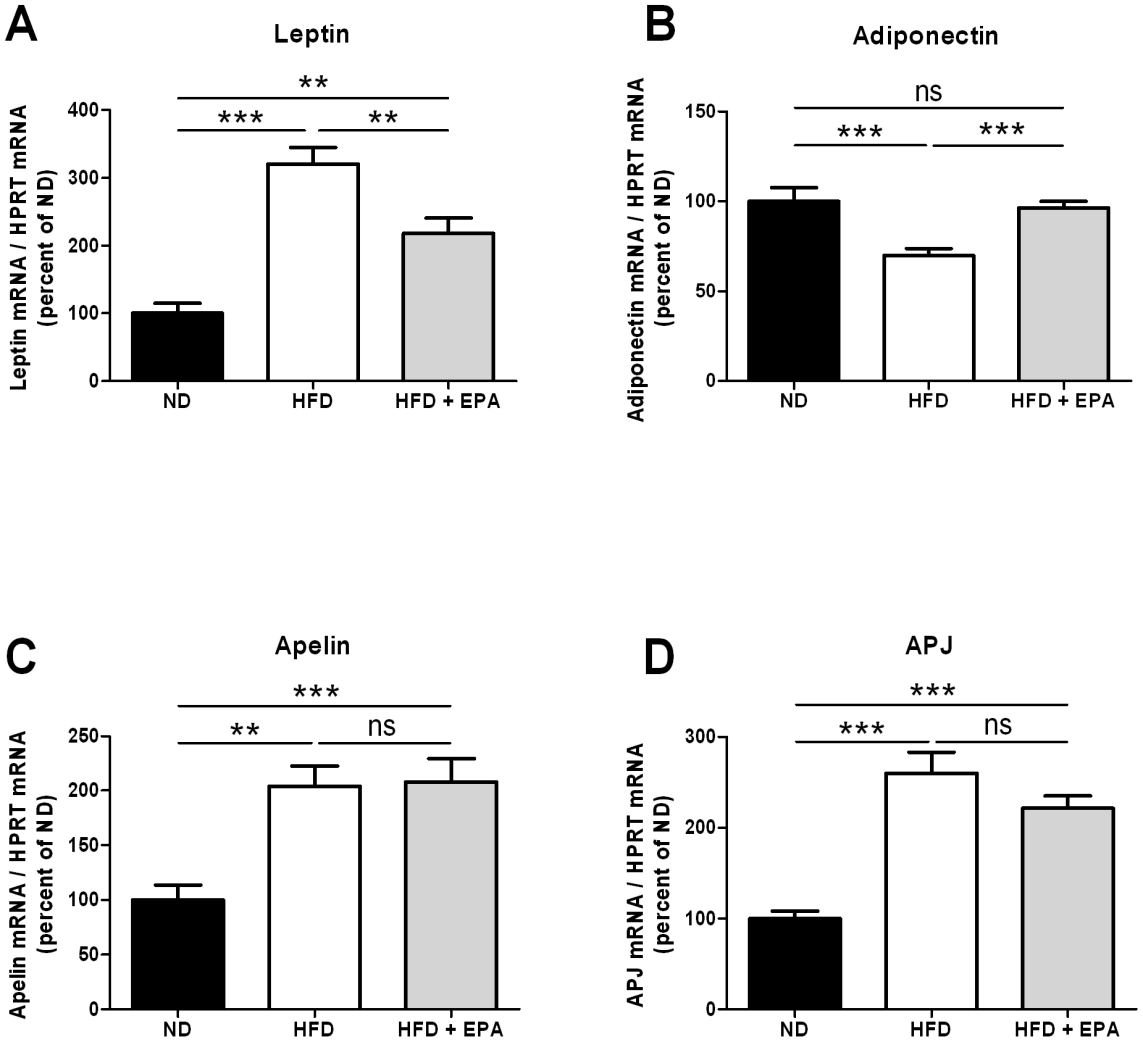
Effect of EPA on adipokines and APJ mRNA expression in adipose tissue. (A) Leptin, (B) Adiponectin, (C) Apelin and (D) APJ expression in total adipose tissue of ND (n = 12), HFD (n = 14) and HFD+EPA mice (n = 14). Results represent mean ±SEM **p<0.01, ***p<0.001

Since HFD+EPA mice had a lower fat mass than HFD mice and an insulinemia similar to control mice, the expression of adipokines such as leptin (associated to fat mass expansion) and adiponectin (associated to insulin sensitivity) were measured in subcutaneous adipose tissue. As expected, leptin was increased and adiponectin expression decreased in adipose tissue of HFD mice compared to ND (Fig.4A, B). In adipose tissue of HFD+EPA mice, leptin expression was decreased whereas adiponectin was increased compared to HFD mice. These results are in agreement with the “healthy” phenotype of HFD+EPA mice described in figure 2 and 3. Concerning the apelin/APJ system, both apelin and APJ mRNA levels were increased in adipose of HFD mice as previously reported [19]. However, their expressions were not different between HFD and HFD+EPA mice in subcutaneous (Fig 4C and D) and perigonadal adipose tissue (not shown). Plasma apelin levels were not modified either (0.70±0.03 ng/ml in HFD mice (n = 14) and 0.67±0.02 ng/ml in HFD+EPA mice (n = 14)).

### Effect of EPA on muscle lipid metabolism

**Figure 5:**
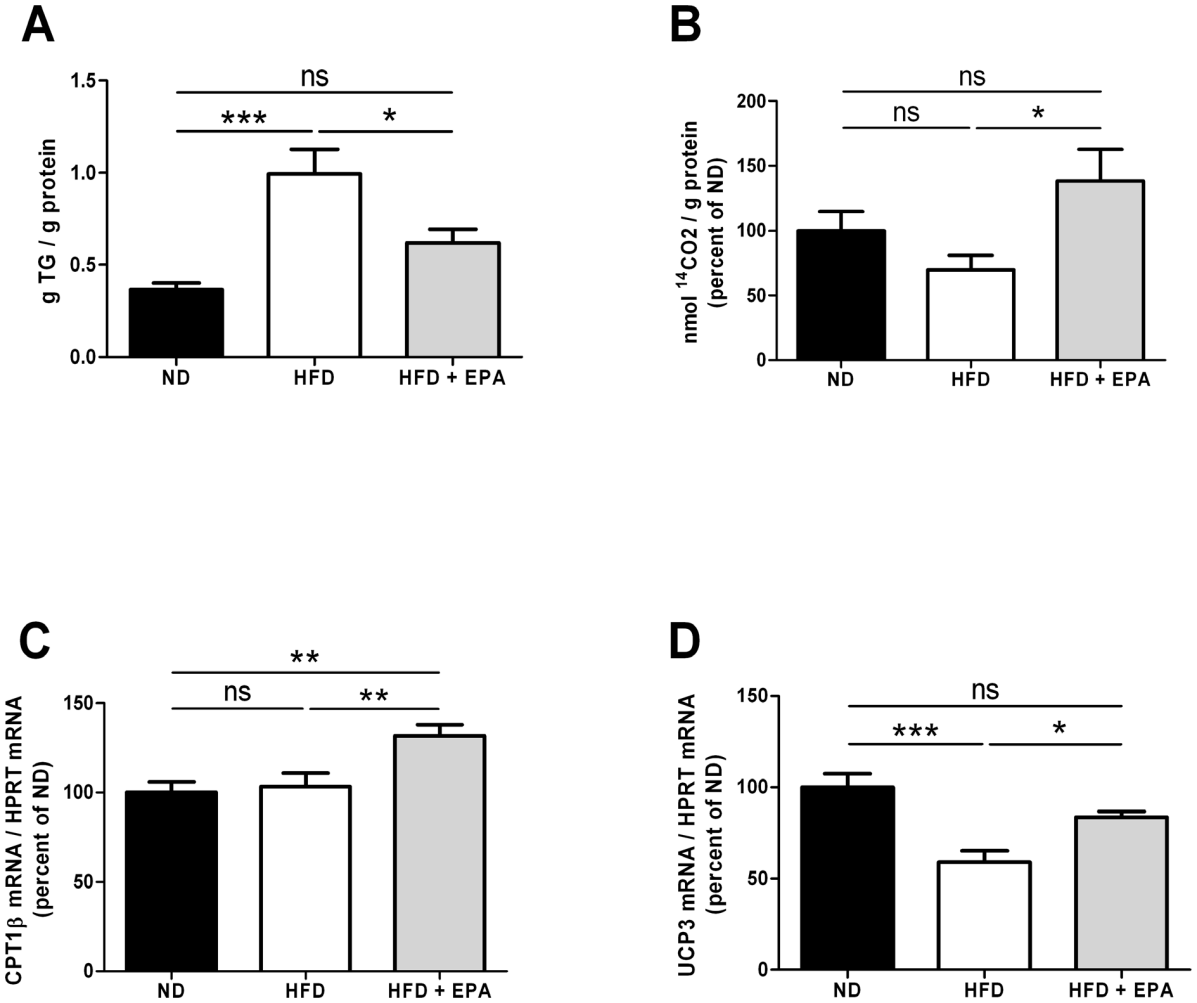
Effect of EPA on muscle lipid metabolism. (A) Intramuscular triglycerides content in red gastrocnemius of ND (n = 9), HFD (n = 10) and HFD+EPA (n = 10) mice. (B) ^14^C-palmitate complete β-oxidation and mRNA expression of (C) CPT1b and (D) UCP3 in soleus muscle of ND (n = 9), HFD (n = 6–10) and HFD+EPA mice (n = 6–10). Results are mean ±SEM and were normalized to the ND group (100%) for B, C and D, *p<0.05, **p<0.01, ***p<0.001

Since muscles are mainly involved in substrate utilization, we focused on lipid utilization by measuring the intramuscular triglycerides (IMTG) and the ^14^C-palmitate oxidation ex vivo in soleus muscle. Muscle homogenates of HFD mice contained more IMTG when compared to HFD+EPA and control mice (Fig. 5A). Moreover the complete oxidation of ^14^C-palmitate to CO_2_ (Fig. 5B), although not significantly decreased in muscle of HFD mice, was significantly increased in muscle of HFD+EPA compared to HFD mice. The expression of carnitine-palmitoyl-transferase 1b (CPT1b), the rate-limiting enzyme in mitochondrial fatty acid oxidation, as well as the uncoupling protein-3 (UCP3), known to increase with muscle mitochondrial content and to enhance FAO [25], were also significantly increased (Fig. 5C, D) in muscle of HFD+EPA compared to HFD. Thus, limited IMTG storage coupled to increased fatty acid oxidation account for a better utilization of lipids and less metabolic alterations in muscle of HFD+EPA mice.

### Effect of EPA on apelin and APJ gene expression in skeletal muscle and C2C12 myotubes

**Figure 6:**
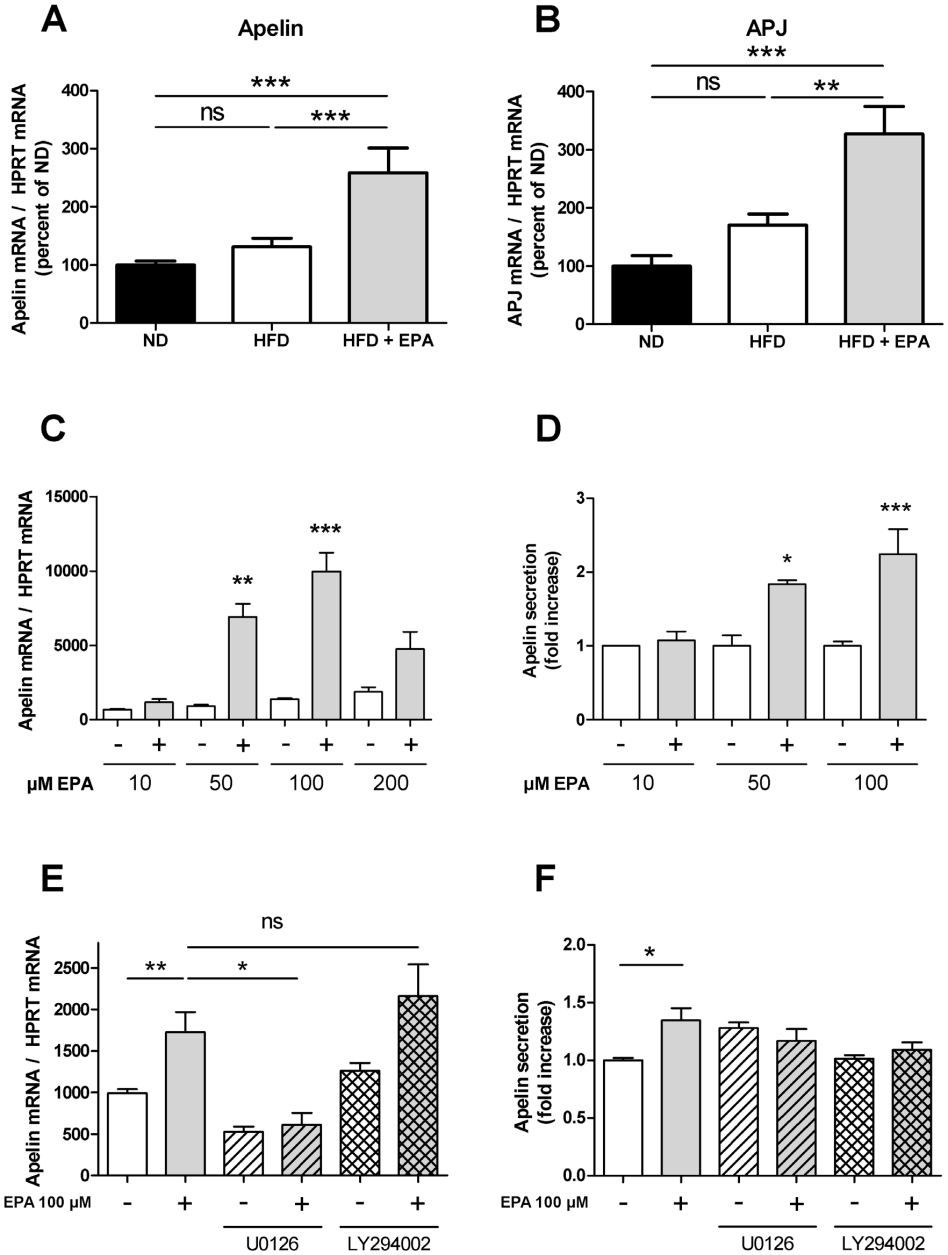
EPA induces in vivo and in vitro expression of the apelin/APJ system in muscle. (A) Apelin and (B) APJ mRNA expression in soleus muscle of ND (n = 7), HFD (n = 10) and HFD+EPA (n = 5). Results represent mean ±SEM **p<0.01, ***p<0.001, ns: not significant. (C) Apelin mRNA expression and (D) secretion in differentiated C2C12 cells treated with the indicated EPA concentrations or the corresponding BSA content for 24 hours after 12-hour serum deprivation. Data are mean ±SEM n = 4–5 in each condition. *p<0.05, **p<0.01 ***p<0.001. (E) Effect of 100 µM EPA in the absence or in the presence of the PI3K inhibitor LY294002 (20 µM) or the ERK 1/2 inhibitor U0126 (20 µM) for 24 h after 12-hour serum deprivation on apelin mRNA expression and (F) secretion in differentiated C2C12 cells. Results represent mean ±SEM (n = 4–5 in each condition) *p<0.05, **p<0.01, ns: not significant.

Apelin and APJ expression in muscle of HFD mice were not significantly increased compared to ND mice as previously reported [19]. However, a significant increase was observed in muscle of HFD+EPA (Fig. 6A and B). In order to confirm a direct effect of EPA on apelin and APJ expression, an in vitro approach on C2C12 muscle cells was performed. Of note, APJ expression was not detectable in C2C12 after 14 days of differentiation (data not shown). However, apelin expression was increased after a 24 h EPA treatment in a dose-dependent manner with a maximal effect at 100 µM EPA (Fig. 6C). Interestingly, EPA not only increased significantly apelin expression but also apelin secretion (Fig. 6D). The values obtained with 100 µM EPA were 19.67±5.00 pg/ml in control condition (n = 4) and 39.80±5.90 pg/ml in response to 100 µM EPA (n = 5). To get further insight, the pathway regulating the stimulatory effect of EPA on apelin gene expression was investigated. Since several peroxisome proliferator activated receptors (PPAR) responsive elements are present in the human promoter of apelin [26] and since PPAR are activated by n-3 PUFAs and PPARα is involved in partitioning fatty acids towards oxidation [27], we measured the expression of PPARα in response to 100 µM EPA in C2C12 myotubes. Even though PPARα gene expression was very weak, no increase was observed in response to EPA (not shown) suggesting that PPARα does not play a major role in the stimulatory effect of EPA on apelin gene expression. Next, since PI3K and MAPK pathways have been shown to regulate insulin or TNFα-induced apelin expression in adipocytes [28], [29], C2C12 cells were treated with the PI3K inhibitor LY 294002 (20 µM) or the ERK 1/2 inhibitor U0126 (20 µM) in the absence or presence of 100 µM EPA. As shown in Fig. 6E, only the treatment with U0126 decreased the stimulatory effect of EPA on apelin gene expression suggesting that the MAPK pathway is involved. However, U0126 had no effect on apelin secretion induced by EPA (Fig.6F) suggesting that this mechanism is not dependent on the ERK activation.

## Discussion

In the present study we investigated in mice the effect of a HFD supplemented with EPA on metabolic disturbances usually associated with HFD feeding. We also studied the regulation of the apelin/APJ system especially in skeletal muscles, since chronic apelin treatment has recently been shown to improve insulin sensitivity mainly by increasing muscle oxidative capacities [20].

The effects of n-3 PUFAs (either a single species or in combination) on obesity have been studied in several animal models. In mice fed a HFD enriched with EPA in a similar amount than in the present study (45% energy from fat, 3,6% EPA), even though the amount given is far beyond the amount that can be given in humans, significant decreased weight gain associated with decreased adiposity was also observed [6]. However the anti-obesity effect of EPA has not been found in mice fed a HFD containing low sucrose (38.1% fat, 8.5%sucrose, 5% EPA), unlike in mice fed a high-fat/high-sucrose diet (25% fat, 32.5%sucrose, 5% EPA) [7]. This underlines the importance of the composition of the diet in both the treated and control group. More recently, attenuated body weight gain and reduced fat mass percentage has also been observed in golden Syrian hamsters fed a HFD supplemented with n-3 PUFA [8]. In the present study, EPA supplementation prevents body weight and fat mass gain in agreement with the anti-obesity effect of EPA. Reduced fat mass also has an impact on adipokines profile in adipose tissue as shown here by a decrease in leptin expression and an increase in adiponectin expression as previously reported [9], [12], [13]. Moreover, significant correlations have been found between obesity and the fatty acid composition in serum. Lipid profile in serum mirrors to a certain extent dietary fatty acid composition and reflects endogenous fatty acid metabolism. For example, high plasma concentrations of palmitic (C16:0) or dihomo-γ-linolenic (C20:3 n-6) acids have been reported to be associated with obesity and the metabolic syndrome in humans [30]. Hereby, plasma concentrations of saturated fatty acids and C20:3 n-6 were decreased in HFD+EPA mice compared to HFD mice, strengthening the beneficial anti-obesity effect of EPA.

Obesity has also been associated with lipid accumulation in the liver leading to steatosis. Limited storage of TG in the liver in response to EPA has been observed in several studies in rodents [7]–[9] but also in humans [31]. This was confirmed in the present study, in mice, by an absence of TG accumulation and no increased expression of SREBP-1c in the liver of HFD+EPA mice compared to HFD mice. Since SREBP-1c controls the expression of lipogenic enzymes, this suggests that a decrease in lipogenesis could contribute to the EPA protective effect from HFD-induced obesity. The weak expression of hepatic SREBP-1c could be due to a direct effect of EPA on SREBP-1c gene transcription as previously reported [32] or related to the low concentration of plasma insulin observed in HFD+EPA mice since this transcription factor is regulated by insulin [33].

EPA effects on adipose tissue and liver and consequently their decrease in lipid content, questioned about the fate of fatty acids. This is why we focused on the study of lipid metabolism in skeletal muscle. Indeed, resistance to obesity could be due to increased fatty acid oxidation in the muscles. This function has rarely been measured in response to EPA in muscle. Hereby, fatty acid oxidation and the expression of CPT1b were increased in soleus muscle of HFD+EPA mice. Thus, the observed increase in CTP1b expression in response to EPA most likely promotes the entry of fat into the mitochondria and subsequent oxidation. A physiological overexpression of CPT1 in skeletal muscle was shown to increase fatty acid oxidation and to prevent HFD-induced fatty acid esterification into intracellular lipids that resulted in enhanced muscle insulin sensitivity [34]. Increased UCP3 expression has also been associated with a better use of lipids. In the present study, UCP3 expression was significantly increased in muscle of HFD+EPA mice. Constitutive UCP3 overexpression at physiological levels (over 2-fold) has been shown to decrease the respiratory exchange ratio and to increase mouse skeletal muscle capacity for fatty acid transport and oxidation [35]. Thus, a better use of lipids balanced by a decrease of IMTG in muscle could contribute to a better insulin sensitivity. In line with this, EPA supplementation in HFD fed mice prevented hyperinsulinemia and hyperglycemia and the impaired glucose tolerance observed in HFD mice.

Like EPA, apelin has been shown to increase insulin sensitivity in obese and insulin-resistant mice [15], [20]. Expression and secretion of apelin in response to EPA treatment has been studied, until now, only in adipose tissue [16], [17]. Since apelin plays a major role in muscle metabolism, it was important to determine whether EPA could regulate apelin expression in muscle comparatively to adipose tissue. The liver is not a major target of apelin since APJ is weakly expressed in basal conditions [36]. To our knowledge, APJ regulation by EPA has never been reported but EPA has been shown to regulate the leptin receptor expression [37]. APJ gene expression in adipose tissue was not differentially regulated between HFD and HFD+EPA fed mice. Similar results were obtained for apelin gene expression although protein levels were not measured. However, apelin mRNA has been shown to parallel protein level in different tissues and situations [38], [39]. The absence of significant increase of apelin expression in adipose tissue of EPA+HFD mice compared to HFD mice differs from the previous published in vivo study [17]. This discrepancy could be due to the composition of the diet, the model used and how EPA was administered. Indeed, Perez-Echarri et al have shown increased apelin expression in adipose tissue of rats fed a high-fat cafeteria diet where EPA was given daily by oral administration [17]. However, since HFD+EPA mice in the present study had less fat mass and lower insulinemia (both factors being able to increase apelin [28]) than HFD mice, a decrease in apelin production might even be expected. Thus, EPA may have an effect on the expression of apelin and APJ in adipose tissue of HFD+EPA fed mice.

We report here for the first time an increase of apelin expression in the muscle of HFD+EPA mice compared to HFD mice and in C2C12 myotubes. The signaling pathway regulating the stimulatory effect of EPA on apelin gene expression in C2C12 myotubes was dependent on ERK1/2 but not PI3K activation. These results are different to those obtained by Lorente-Cebrian et al showing that in 3T3-L1 adipocytes, EPA-induced apelin gene expression was dependent of PI3K and independent of MAPK activation [16]. However, the pharmacological inhibitors and the cell type used were not the same than in the present study. Moreover, U0126, the ERK1/2 inhibitor was unable to inhibit the stimulatory effect of EPA on apelin secretion. A difference between apelin gene expression and apelin secretion in response to an inhibitor has been already reported in adipocytes [16]. These results suggest that the mechanism involved in apelin secretion might be MAPK independent. Further investigations are necessary to better define the different signaling pathways and whether other targets are involved. Moreover, EPA effect on apelin secretion seems to be more moderate that the effect observed on apelin gene expression underlying that indeed different mechanism could be involved. However it could not be excluded that apelin could be stored like in cardiomyocytes [40] and only a part of the produced apelin, is secreted. All together, these results suggest that a direct effect of EPA on the expression of apelin in vivo could be expected. Additionally and importantly, EPA in differentiated C2C12 efficiently stimulates not only apelin gene expression but also apelin secretion. Thus, apelin could act as a paracrine or autocrine factor in the muscle and could be then considered as a new beneficial myokine.

In conclusion, dietary EPA prevents obesity and metabolic alterations in mice fed a HFD. However, careful attention is necessary when extrapolating to human interventions since, very recently, too high n3-PUFAs plasma levels were shown to correlate with increased prostate cancer [41]. In the present study, in skeletal muscle of HFD+EPA mice, the apelin/APJ system was up-regulated, a better use of lipids was shown matching with the decreased steatosis and decreased fat mass. By combining the results obtained in vitro and in vivo, it could be hypothesized that in response to EPA, increased APJ expression in muscle and increased apelin production (expression and secretion), could, in turn, contribute to improved muscle metabolism by a paracrine/autocrine regulation loop.

## References

[1] Kopecky J, Rossmeisl M, Flachs P, Kuda O, Brauner P, et al (2009) n-3 PUFA: bioavailability and modulation of adipose tissue function. Proc Nutr Soc 68: 361–369. 19698199 10.1017/S0029665109990231

[2] Delarue J, LeFoll C, Corporeau C, Lucas D (2004) N-3 long chain polyunsaturated fatty acids: a nutritional tool to prevent insulin resistance associated to type 2 diabetes and obesity? Reprod Nutr Dev 44: 289–299. 15460168 10.1051/rnd:2004033

[3] Siriwardhana N, Kalupahana NS, Moustaid-Moussa N (2012) Health benefits of n-3 polyunsaturated fatty acids: eicosapentaenoic acid and docosahexaenoic acid. Adv Food Nutr Res 65: 211–222. 22361189 10.1016/B978-0-12-416003-3.00013-5

[4] Fedor D, Kelley DS (2009) Prevention of insulin resistance by n-3 polyunsaturated fatty acids. Curr Opin Clin Nutr Metab Care 12: 138–146. 19202385 10.1097/MCO.0b013e3283218299

[5] Poudyal H, Panchal SK, Diwan V, Brown L (2011) Omega-3 fatty acids and metabolic syndrome: effects and emerging mechanisms of action. Prog Lipid Res 50: 372–387. 21762726 10.1016/j.plipres.2011.06.003

[6] Kalupahana NS, Claycombe K, Newman SJ, Stewart T, Siriwardhana N, et al (2010) Eicosapentaenoic acid prevents and reverses insulin resistance in high-fat diet-induced obese mice via modulation of adipose tissue inflammation. J Nutr 140: 1915–1922. 20861209 10.3945/jn.110.125732

[7] Sato A, Kawano H, Notsu T, Ohta M, Nakakuki M, et al (2010) Antiobesity effect of eicosapentaenoic acid in high-fat/high-sucrose diet-induced obesity: importance of hepatic lipogenesis. Diabetes 59: 2495–2504. 20682690 10.2337/db09-1554 PMC3279525

[8] Kasbi Chadli F, Andre A, Prieur X, Loirand G, Meynier A, et al. (2011) n-3 PUFA prevent metabolic disturbances associated with obesity and improve endothelial function in golden Syrian hamsters fed with a high-fat diet. Br J Nutr:1–11. 10.1017/S0007114511004387 21920060

[9] Gonzalez-Periz A, Horrillo R, Ferre N, Gronert K, Dong B, et al (2009) Obesity-induced insulin resistance and hepatic steatosis are alleviated by omega-3 fatty acids: a role for resolvins and protectins. FASEB J 23: 1946–1957. 19211925 10.1096/fj.08-125674 PMC2698663

[10] Perez-Matute P, Marti A, Martinez JA, Fernandez-Otero MP, Stanhope KL, et al (2005) Eicosapentaenoic fatty acid increases leptin secretion from primary cultured rat adipocytes: role of glucose metabolism. Am J Physiol Regul Integr Comp Physiol 288: R1682–1688. 15650121 10.1152/ajpregu.00727.2004

[11] Murata M, Kaji H, Takahashi Y, Iida K, Mizuno I, et al (2000) Stimulation by eicosapentaenoic acids of leptin mRNA expression and its secretion in mouse 3T3-L1 adipocytes in vitro. Biochem Biophys Res Commun 270: 343–348. 10753628 10.1006/bbrc.2000.2424

[12] Reseland JE, Haugen F, Hollung K, Solvoll K, Halvorsen B, et al (2001) Reduction of leptin gene expression by dietary polyunsaturated fatty acids. J Lipid Res 42: 743–50. 11352981

[13] Flachs P, Mohamed-Ali V, Horakova O, Rossmeisl M, Hosseinzadeh-Attar MJ, et al (2006) Polyunsaturated fatty acids of marine origin induce adiponectin in mice fed a high-fat diet. Diabetologia 49: 394–397. 16397791 10.1007/s00125-005-0053-y

[14] Tishinsky JM, Ma DW, Robinson LE (2011) Eicosapentaenoic acid and rosiglitazone increase adiponectin in an additive and PPARgamma-dependent manner in human adipocytes. Obesity (Silver Spring) 19: 262–268. 20814411 10.1038/oby.2010.186

[15] Dray C, Knauf C, Daviaud D, Waget A, Boucher J, et al (2008) Apelin stimulates glucose utilization in normal and obese insulin-resistant mice. Cell Metab 8: 437–445. 19046574 10.1016/j.cmet.2008.10.003

[16] Lorente-Cebrian S, Bustos M, Marti A, Martinez JA, Moreno-Aliaga MJ (2010) Eicosapentaenoic acid up-regulates apelin secretion and gene expression in 3T3-L1 adipocytes. Mol Nutr Food Res 54 Suppl 1S104–11. 20352620 10.1002/mnfr.200900522

[17] Perez-Echarri N, Perez-Matute P, Marcos-Gomez B, Martinez JA, Moreno-Aliaga MJ (2009) Effects of eicosapentaenoic acid ethyl ester on visfatin and apelin in lean and overweight (cafeteria diet-fed) rats. Br J Nutr 101: 1059–1067. 18755047 10.1017/S0007114508048307

[18] O'Carroll AM, Selby TL, Palkovits M, Lolait SJ (2000) Distribution of mRNA encoding B78/apj, the rat homologue of the human APJ receptor, and its endogenous ligand apelin in brain and peripheral tissues. Biochim Biophys Acta 1492: 72–80. 11004481 10.1016/s0167-4781(00)00072-5

[19] Dray C, Debard C, Jager J, Disse E, Daviaud D, et al (2010) Apelin and APJ regulation in adipose tissue and skeletal muscle of type 2 diabetic mice and humans. Am J Physiol Endocrinol Metab 298: E1161–1169. 20233941 10.1152/ajpendo.00598.2009

[20] Attane C, Foussal C, Le Gonidec S, Benani A, Daviaud D, et al (2012) Apelin treatment increases complete Fatty Acid oxidation, mitochondrial oxidative capacity, and biogenesis in muscle of insulin-resistant mice. Diabetes 61: 310–320. 22210322 10.2337/db11-0100 PMC3266414

[21] Yamamoto T, Habata Y, Matsumoto Y, Yasuhara Y, Hashimoto T, et al (2011) Apelin-transgenic mice exhibit a resistance against diet-induced obesity by increasing vascular mass and mitochondrial biogenesis in skeletal muscle. Biochim Biophys Acta 1810: 853–862. 21609753 10.1016/j.bbagen.2011.05.004

[22] Lillington JM, Trafford DJ, Makin HL (1981) A rapid and simple method for the esterification of fatty acids and steroid carboxylic acids prior to gas-liquid chromatography. Clin Chim Acta 111: 91–98. 7226543 10.1016/0009-8981(81)90425-3

[23] Folch J, Lees M, Sloane Stanley GH (1957) A simple method for the isolation and purification of total lipides from animal tissues. J Biol Chem 226: 497–509. 13428781

[24] Figueras M, Olivan M, Busquets S, Lopez-Soriano FJ, Argiles JM (2011) Effects of eicosapentaenoic acid (EPA) treatment on insulin sensitivity in an animal model of diabetes: improvement of the inflammatory status. Obesity (Silver Spring) 19: 362–369. 20885391 10.1038/oby.2010.194

[25] Jones TE, Baar K, Ojuka E, Chen M, Holloszy JO (2003) Exercise induces an increase in muscle UCP3 as a component of the increase in mitochondrial biogenesis. Am J Physiol Endocrinol Metab 284: E96–101. 12388129 10.1152/ajpendo.00316.2002

[26] Mazzucotelli A, Ribet C, Castan-Laurell I, Daviaud D, Guigné C, et al (2008) The transcriptional co-activator PGC1alpha up regulates apelin in human and mouse adipocytes. Regul Pept 150: 33–37. 18501443 10.1016/j.regpep.2008.04.003

[27] Calder PC (2012) Mechanisms of action of (n-3) fatty acids. J Nutr 142: 592S–599S. 22279140 10.3945/jn.111.155259

[28] Boucher J, Masri B, Daviaud D, Gesta S, Guigne C, et al (2005) Apelin, a newly identified adipokine up-regulated by insulin and obesity. Endocrinology 146: 1764–1771. 15677759 10.1210/en.2004-1427

[29] Daviaud D, Boucher J, Gesta S, Dray C, Guigne C, et al (2006) TNFalpha up-regulates apelin expression in human and mouse adipose tissue. FASEB J 20: 1528–30. 16723381 10.1096/fj.05-5243fje

[30] Warensjo E, Ohrvall M, Vessby B (2006) Fatty acid composition and estimated desaturase activities are associated with obesity and lifestyle variables in men and women. Nutr Metab Cardiovasc Dis 16: 128–136. 16487913 10.1016/j.numecd.2005.06.001

[31] Di Minno MN, Russolillo A, Lupoli R, Ambrosino P, Di Minno A, et al (2012) Omega-3 fatty acids for the treatment of non-alcoholic fatty liver disease. World J Gastroenterol 18: 5839–5847. 23139599 10.3748/wjg.v18.i41.5839 PMC3491590

[32] Jump DB (2008) N-3 polyunsaturated fatty acid regulation of hepatic gene transcription. Curr Opin Lipidol 19: 242–247. 18460914 10.1097/MOL.0b013e3282ffaf6a PMC2764370

[33] Dentin R, Denechaud PD, Benhamed F, Girard J, Postic C (2006) Hepatic gene regulation by glucose and polyunsaturated fatty acids: a role for ChREBP. J Nutr 136: 1145–1149. 16614395 10.1093/jn/136.5.1145

[34] Bruce CR, Hoy AJ, Turner N, Watt MJ, Allen TL, et al (2009) Overexpression of carnitine palmitoyltransferase-1 in skeletal muscle is sufficient to enhance fatty acid oxidation and improve high-fat diet-induced insulin resistance. Diabetes 58: 550–558. 19073774 10.2337/db08-1078 PMC2646053

[35] Bezaire V, Spriet LL, Campbell S, Sabet N, Gerrits M, et al (2005) Constitutive UCP3 overexpression at physiological levels increases mouse skeletal muscle capacity for fatty acid transport and oxidation. FASEB J 19: 977–979. 15814607 10.1096/fj.04-2765fje

[36] Pope GR, Roberts EM, Lolait SJ, O'Carroll AM (2012) Central and peripheral apelin receptor distribution in the mouse: species differences with rat. Peptides 33: 139–148. 22197493 10.1016/j.peptides.2011.12.005 PMC3314948

[37] Fan C, Liu X, Shen W, Deckelbaum RJ, Qi K (2011) The Regulation of Leptin, Leptin Receptor and Pro-opiomelanocortin Expression by N-3 PUFAs in Diet-Induced Obese Mice Is Not Related to the Methylation of Their Promoters. Nutr Metab (Lond) 8: 31. 21609458 10.1186/1743-7075-8-31 PMC3117679

[38] Wang XL, Tao Y, Lu Q, Jiang YR (2012) Apelin supports primary rat retinal müller cells under chemical hypoxia and glucose deprivation. Peptides 33: 298–306. 22240274 10.1016/j.peptides.2011.12.015

[39] Berta J, Kenessey I, Dobos J, Tovari J, Klepetko W (2010) Apelin expression in human non-small cell lung cancer: role in angiogenesis and prognosis. J Thorac Oncol 8: 1120–1129. 10.1097/JTO.0b013e3181e2c1ff 20581707

[40] Ronkainen VP, Ronkainen JJ, Hänninen SL, Leskinen H, Ruas JL, et al (2007) Hypoxia inducible factor regulates the cardiac expression and secretion of apelin. FASEB J 21: 1821–1830. 17341685 10.1096/fj.06-7294com

[41] Brasky TM, Darke AK, Song X, Tangen CM, Goodman PJ, et al (2013) Plasma Phospholipid Fatty Acids and Prostate Cancer Risk in the SELECT Trial. J Natl Cancer Inst. 105(15): 1132–1141. 23843441 10.1093/jnci/djt174 PMC3735464

